# Supplementary material for: Resolving the full spectrum of human genome variation using Linked-Reads
Source: Genome Res. 2019 Apr;29(4):635–45. doi: 10.1101/gr.234443.118 (PMC6442396; doi:10.1101/gr.234443.118)
Supplement: Supplemental Material [file supp_gr.234443.118_Supplemental_longranger-2.2.zip › longranger-2.2.tar.bz2!/lr2.2-public/lib/stan/cmdstan/stan/doc/api/html/rev_2operators_2operator__division_8hpp_source.html]

Stan: src/stan/agrad/rev/operators/operator\_division.hpp Source File


|  |  |
| --- | --- |
|  | Stan  2.5.0  probability, sampling & optimization |


- Main Page
- Related Pages
- Namespaces
- Classes
- Files

- File List
- File Members

All Classes Namespaces Files Functions Variables Typedefs Enumerations Enumerator Friends Macros Pages

- src
- stan
- agrad
- rev
- operators

operator\_division.hpp

Go to the documentation of this file.

1 #ifndef STAN\_\_AGRAD\_\_REV\_\_OPERATORS\_\_OPERATOR\_DIVISION\_HPP

2 #define STAN\_\_AGRAD\_\_REV\_\_OPERATORS\_\_OPERATOR\_DIVISION\_HPP

3

4 #include <stan/agrad/rev/var.hpp>

5 #include <stan/agrad/rev/internal/vv\_vari.hpp>

6 #include <stan/agrad/rev/internal/vd\_vari.hpp>

7 #include <stan/agrad/rev/internal/dv\_vari.hpp>

8 #include <boost/math/special\_functions/fpclassify.hpp>

9

10 namespace stan {

11  namespace agrad {

12

13  namespace {

14  // (a/b)' = a' \* (1 / b) - b' \* (a / [b \* b])

15  class divide\_vv\_vari : public op\_vv\_vari {

16  public:

17  divide\_vv\_vari(vari\* avi, vari\* bvi) :

18  op\_vv\_vari(avi->val\_ / bvi->val\_, avi, bvi) {

19  }

20  void chain() {

21  if (unlikely(boost::math::isnan(avi\_->val\_)

22  || boost::math::isnan(bvi\_->val\_))) {

23  avi\_->adj\_ = std::numeric\_limits<double>::quiet\_NaN();

24  bvi\_->adj\_ = std::numeric\_limits<double>::quiet\_NaN();

25  } else {

26  avi\_->adj\_ += adj\_ / bvi\_->val\_;

27  bvi\_->adj\_ -= adj\_ \* avi\_->val\_ / (bvi\_->val\_ \* bvi\_->val\_);

28  }

29  }

30  };

31

32  class divide\_vd\_vari : public op\_vd\_vari {

33  public:

34  divide\_vd\_vari(vari\* avi, double b) :

35  op\_vd\_vari(avi->val\_ / b, avi, b) {

36  }

37  void chain() {

38  if (unlikely(boost::math::isnan(avi\_->val\_)

39  || boost::math::isnan(bd\_)))

40  avi\_->adj\_ = std::numeric\_limits<double>::quiet\_NaN();

41  else

42  avi\_->adj\_ += adj\_ / bd\_;

43  }

44  };

45

46  class divide\_dv\_vari : public op\_dv\_vari {

47  public:

48  divide\_dv\_vari(double a, vari\* bvi) :

49  op\_dv\_vari(a / bvi->val\_, a, bvi) {

50  }

51  void chain() {

52  bvi\_->adj\_ -= adj\_ \* ad\_ / (bvi\_->val\_ \* bvi\_->val\_);

53  }

54  };

55  }

56

95  inline var operator/(const var& a, const var& b) {

96  return var(new divide\_vv\_vari(a.vi\_,b.vi\_));

97  }

98

110  inline var operator/(const var& a, const double b) {

111  if (b == 1.0)

112  return a;

113  return var(new divide\_vd\_vari(a.vi\_,b));

114  }

115

127  inline var operator/(const double a, const var& b) {

128  return var(new divide\_dv\_vari(a,b.vi\_));

129  }

130

131  }

132 }

133 #endif

boost::math::isnan

bool isnan(const stan::agrad::var &v)

Checks if the given number is NaN.

**Definition:** boost\_fpclassify.hpp:69

dv\_vari.hpp

stan::agrad::operator/

Eigen::Matrix< fvar< T >, R, C > operator/(const Eigen::Matrix< fvar< T >, R, C > &v, const fvar< T > &c)

**Definition:** divide.hpp:58

vd\_vari.hpp

stan::agrad::var::vi\_

vari \* vi\_

Pointer to the implementation of this variable.

**Definition:** var.hpp:40

unlikely

#define unlikely(x)

**Definition:** likely.hpp:9

var.hpp

stan::agrad::var

Independent (input) and dependent (output) variables for gradients.

**Definition:** var.hpp:27

vv\_vari.hpp


---

[ Stan Home Page ]

*© 2011–2014,
Stan Development Team.*
